# INTERNET ADDICTION AMONG ADOLESCENTS IN A WESTERN BRAZILIAN AMAZONIAN CITY

**DOI:** 10.1590/1984-0462/2021/39/2019270

**Published:** 2020-07-03

**Authors:** Tatiane Dalamaria, Wagner de Jesus Pinto, Edson dos Santos Farias, Orivaldo Florencio de Souza

**Affiliations:** a Universidade Federal do Acre, Rio Branco, AC, Brazil.; b Universidade Federal de Rondônia, Porto Velho, RO, Brazil.

**Keywords:** Addictive behavior, Leisure activities, Internet, Adolescents, Comportamento aditivo, Atividades de lazer, Internet, Adolescente

## Abstract

**Objective:**

Analyze the prevalence and factors associated with internet addiction in a sample of high school adolescents in Acre, a state in northern Brazil.

**Methods:**

A population-based cross-sectional study was conducted with 1,387 adolescents between 14 and 18 years old enrolled in high schools in the city of Rio Branco, Acre. A structured questionnaire with questions about demographics, family and behavioral patterns was applied. Internet addiction was verified using the Internet Addiction Test (IAT), with a cutoff equal to or greater than 70 points. The associated factors were identified by multiple logistic regression analyses.

**Results:**

The overall prevalence of Internet addiction was 10.6%. Higher dependence was observed in females. The factors associated with Internet addiction were being female, using the computer for more than two hours a day during weekdays and on the weekend, not practicing physical activities and going out to dance at nightclubs and concerts at least once a month. A protective association of reading habits in relation to Internet dependence was observed.

**Conclusions:**

The present study showed a high prevalence of Internet dependence, with female adolescents being more susceptible. Behavioral aspects were associated with internet dependence in a sample of adolescents from Rio Branco, Acre.

## RESUMO

**Objetivo:**

Analisar a prevalência e os fatores associados à dependência de internet em uma amostra de adolescentes do ensino médio no Acre.

**Métodos:**

Estudo transversal de base populacional realizado com 1.387 adolescentes entre 14 e 18 anos regularmente matriculados em instituições de ensino médio no município de Rio Branco, Acre. Questionário estruturado com questões sobre aspectos demográficos, sociais, familiares e comportamentais foi aplicado. A dependência de internet foi verificada pelo *internet addiction test* (IAT), com ponto de corte igual ou superior a 70. Os fatores associados foram identificados mediante regressão logística múltipla.

**Resultados:**

A prevalência geral para dependência de internet foi de 10,6%. Maior dependência foi evidenciada no sexo feminino. Os fatores associados à dependência de internet foram ser do sexo feminino, utilizar o computador por mais de duas horas diárias durante o meio de semana e no fim de semana, não praticar atividade física e sair por pelo menos uma vez ao mês para dançar em boates e *shows*. Observou-se associação protetora do hábito de leitura em relação à dependência de internet.

**Conclusões:**

O presente estudo evidenciou alta prevalência para dependência de internet, sendo as adolescentes do sexo feminino mais suscetíveis. Aspectos comportamentais estiveram associados à dependência de internet em uma amostra de adolescentes de Rio Branco.

## INTRODUCTION

Nowadays, the internet is an essential part of everyday life, as it enables the fluidity necessary for the circulation of information and global communication.^1^ ^,^ ^2^ Although there are benefits to this, a new pathology called internet addiction has occurred.^1^ ^,^ ^3^ ^,^ ^4^ As such, a fine line separates essential access to work, the teaching-learning process, social communication, and information searching with the compulsive and pathological use of the internet. Therefore, internet dependency is a new and growing problem in public health, requiring greater understanding by the scientific community in order to propose community interventions.

Several factors are associated with internet addiction, such as sociodemographic aspects related to internet use and psychosocial habits^5^ ^,^ ^6^ ^,^ ^7^. Some studies have reported that types of leisure activities done in one’s free time, in addition to the multiple activities performed online, are predisposing factors for internet dependence.^8^ ^,^ ^9^ Specifically, systematic reviews have indicated several adverse effects of internet addiction on mental health, such as attention deficit hyperactivity disorder, depression, hostility and low self-esteem.^1^ ^,^ ^5^ ^,^ ^6^ ^.^ ^7^

Data from the Brazilian National Household Sample Survey indicate that, in Brazil, 49.4% of people are connected to the internet, and this is mostly concentrated in the 15-17-year-old age group.^10^ Thus, the aim of this study was to investigate the prevalence of internet addiction and associated factors in high school students in the municipality of Rio Branco, in the Brazilian state of Acre.

## METHOD

This is a population-based, cross-sectional study with high school students in the municipality of Rio Branco, conducted in the months of May and June 2015. The study population consisted of 20,476 students enrolled in 37 high schools in the urban area of Rio Branco. To determine sample size, an expected prevalence for internet dependency of 12% was considered, as observed in high school students in China.^8^ Precision was set for a sampling error of 0.03 and a level of confidence of 95% was set. With these criteria, the study required a sample of 1,182 students. To protect the investigation from the effects of non-response, 18% was added to the sample size, taking into account operational restrictions, such as the number of researchers and the time available for data collection. The final sample size was estimated at 1,391 students.

The selection of students took place in three stages. First, five schools were selected by simple random sampling. Three schools were public and two were private. This number of schools was based on operational restrictions for data collection. In the second stage, classrooms from each school were chosen by allocation proportional to size, and students were chosen by classroom in the third stage. As inclusion criteria, students aged between 14 and 18 years and 11 months, who were regularly enrolled in a high school institution in the city of Rio Branco were considered. The exclusion criteria were pathologies that would make it impossible for students to participate in data collection without assistance, given the importance of privacy in answering the questions. The study was approved by the Research Ethics Committee of the Universidade Federal do Acre under protocol number 39594914.8.0000.5010. Before beginning data collection, the parents and adolescents signed a free and informed consent form.

A structured and self-answered questionnaire was applied to students with questions regarding demographic (gender and age), social and family (marital status, being an only child, number of true friends and number of teenagers at home), leisure activities (physical activity; going to the movies and theater, going out to dance at nightclubs and shows; and reading newspapers, magazines or books), parental control and computer usage time.

To obtain information on physical activity, the self-administered physical activity checklist was applied, and then validated by Farias Junior et al.^11^ Using this instrument, the types of physical activity and the number of minutes engaging in physical activity per week were verified. This variable was categorized into: equal to or greater than 300 minutes, between 150 and 299 minutes, from 1 and 149 minutes, and does not practice physical activity.

According to the protocol suggested by Petroski,^12^ body weight was measured using a digital portable scale with a maximum capacity of 150 kg and a sensitivity of 50 g, while height was measured using a portable stadiometer with a maximum extension of 2 subdivided into 0.1 cm. The variable body mass index (BMI) was used as recommended by the World Health Organization,^13^ with excess weight (sum of overweight and obese individuals) determined using BMI values for age equal to or greater than 1 Z score.

The test for internet addiction was verified by the internet addiction test (IAT),^14^ which was validated and adapted into the Portuguese language.^2^ ^,^ ^15^ The test consists of 20 questions on a Likert scale ranging from 1 (rarely) to 5 (always), for the individuals to fill out themselves. Individuals with a score equal to or greater than 70 points were considered to be internet dependent.

Statistical analysis of the data was performed using the Stata program, version 10 (Stata Corp. College Station, TX, United States). The associated factors were identified in two stages. First, the independent variables that showed associations with internet dependence with p≤0.20 by the Wald test in simple logistic regression were selected to compose the final multiple model. Subsequently, using multiple logistic regression, using the step-by-step procedure with retrograde elimination, variables with p≤0.05 were selected to compose the final multiple model. The variables with a -value between 0.05 and 0.10 remained in the model as adjustment variables.

## RESULTS

Of the 1,391 adolescents contacted, there were four losses due to refusal to participate in the study. Thus, 1,387 adolescents were considered in the analysis, of which 76.8% were from public schools and 23.2% were from private schools. Of these, 53.1% were female, 44.5% were between 14 and 15 years old, and 55.5% were between 16 and 18 years old.

The overall prevalence of internet addiction was 10.6%. Male students showed a lower prevalence (6.9%) of internet dependence in contrast to female students (13.9%). However, no statistical difference was identified between the ages for internet dependence.

The variables sex, true friends, use of a computer in the bedroom, times using a computer in the middle of the week and on the weekend, going to the movies or theater, going out to dance in nightclubs or *shows*, reading the newspaper, magazines or books, and practicing physical activity were candidates for the final multiple model (Tables 1 and 2).

**Table 1:** Prevalence (%) and Odds Ratio of internet dependence according to demographic, social and family variables and body mass index in high school adolescents, Rio Branco, Acre, Brazil.

	n*	%	OR	p-value
Sex				
Male	650	6.9	1	
Female	737	13.9	2.18	<0.001
Age				
14-15 years	616	11.2	1	
16-18 years	771	10.2	0.90	0.567
Only child				
No	1,199	10.5	1	
Yes	169	11.2	1.07	0.772
True friends				
6 or more friends	411	8.5	1	
1 to 5 friends	781	11.9	1.45	0.073
No Friends	77	12.9	1.60	0.217
Another teenager at home				
Yes	844	10.0	1	
No	515	11.4	1.15	0.421
BMI				
Eutrophic	1,046	10.5	1	
Overweight	304	11.5	1.10	0.621

*The total n does not add up to 1,387 for all variables because some questions did not have answers.

OR: Odds Ratio; BMI: body mass index.

**Table 2:** Prevalence (%) and Odds Ratio of internet dependence according to variables of leisure activities, parental control and time spent using computers in high school adolescents, Rio Branco, Acre, Brazil.

	n*	%	OR	p-value
Using a computer in their room				
No	1,041	9.1	1	
Yes	331	15.7	1.85	0.001
Parental control over computer use				
Yes	522	9.9	1	
No	852	11.0	1.12	0.532
Computer time during the week				
Up to two hours a day	1,329	9.8	1	
More than two hours a day	58	29.3	3.79	<0.001
Computer time during the weekend				
Up to two hours a day	1,077	8.4	1	
More than two hours a day	310	18.3	2.44	<0.001
Going to the movies or theater				
Rarely or never	549	11.1	1	
Monthly	619	8.5	0.74	0.144
Weekly	206	15.0	1.41	0.142
Going out to dance at nightclubs or shows				
Rarely or never	974	8.2	1	
Monthly	247	12.9	1.66	0.022
Weekly	138	22.4	3.23	<0.001
Reading newspapers, magazines or books				
Rarely or never	419	12.6	1	
Monthly	314	11.4	0.89	0.627
Weekly	641	8.7	0.66	0.041
Physical activity				
> 300 minutes	591	7.9	1	
150 to 299 minutes	188	8.5	1.07	0.807
1 to 149 minutes	197	10.6	1.38	0.243
Does not practice physical activity	323	17.0	2.37	<0.001

*The total n does not add up to 1,387 for all variables because some questions did not have answers.

OR: Odds Ratio.

In the analysis of factors associated with internet dependence (Table 3), girls were 1.84 times more likely to be dependent on the internet than boys. Adolescents who spent more than two hours on the computer on weekdays and on the weekend showed associations of 2.39 and 2.08, respectively, with regard to internet dependence. Adolescents who did not practice physical activity were 2.27 times more likely to be dependent on the internet when compared to those who practiced more than 300 minutes per week, while adolescents who went out weekly to dance at nightclubs or shows were 3.32 times more likely to be dependent on the Internet than those who rarely or never frequented such places. The habit of reading weekly was presented as an adjustment variable in the final model of internet dependence.

**Table 3:** Multiple model of internet addiction in high school adolescents, Rio Branco, Acre, Brazil, described in Odds Ratio and 95% confidence interval.

	OR	95%CI	p-value
Sex			
Male	1		
Female	1.84	1.21 - 2.80	0.004
Computer time during the week			
Up to two hours a day	1		
More than two hours a day	2.39	1.18 - 4.85	0.015
Computer time during the weekend			
Up to two hours a day	1		
More than two hours a day	2.08	1.36 - 3.18	0.001
Physical activity			
> 300 minutes	1		
150 to 299 minutes	1.21	0.64 - 2.30	0.543
1 to 149 minutes	1.24	0.68 - 2.28	0.469
Does not practice physical activity	2.27	1.42 - 3.64	0.001
Reading newspapers, magazines or books			
Rarely or never	1		
Monthly	1.08	0.66 - 1.77	0.744
Weekly	0.69	0.44 - 1.07	0.099
Going out to dance in nightclubs or *shows*			
Rarely or never	1		
Monthly	1.74	1.08 - 2.79	0.021
Weekly	3.32	1.99 - 5.56	<0.001

OR: Odds Ratio; 95%CI: 95% confidence interval.

## DISCUSSION

In this cross-sectional population-based study in the Brazilian Amazon region with high school teenagers, a prevalence of 10.6% internet dependence was identified. Girls showed a higher prevalence of dependence in contrast to boys. The factors associated with internet dependency were use of a computer in free time for more than two hours a day, physical inactivity, and going out to dance in nightclubs and shows.

The high prevalence of internet addiction observed here may have been stimulated by the digital inclusion policy, which made free internet access available in various public places in the city of Rio Branco, including schools. Kuss et al.^16^ suggest that unrestricted accessibility to the internet may contribute to internet dependence.

Investigations on internet addiction have shown different prevalence levels in several countries. This variability in prevalence may be due to the numerous measurement instruments and cutoff points used to demarcate levels of dependence. In addition, the inequality in access to the internet in various locations, due to cultural diversity, may have contributed to this variation in the prevalence of internet dependence.^17^ Therefore, caution should be used when comparing the prevalence in studies with different methods. However, considering only research that used a cutoff point equal to or greater than 70 points in the IAT, the prevalence of internet dependence verified in the present study was higher in relation to that of students in South Korea (2.3%)^18^ and Lebanon (4.2%).^19^

In modeling the associated factors, the female gender presented a greater association with internet dependence. Controversially, other studies show that boys are more dependent than girls.^20^ ^,^ ^21^ ^,^ ^22^ However, the level of internet use among females has increased in recent years.^23^

In the present study, the time spent on a computer both during the week and over the weekend showed an association with internet dependence. Analogous information was verified in the city of Shanghai, with respect to the hours spent online on weekdays and weekends.^8^ According to studies in China and Turkey, adolescents who spent more than two hours a day on a computer were more likely to be dependent on the internet compared to those who spent less time.^9^ ^,^ ^24^ Thus, spending more than two hours a day on a computer with Internet access is considered to be sedentary behavior and has the potential to have a negative impact on health.^25^ Therefore, controlling adolescents’ time spent on computers is an effective method for preventing internet addiction.

Engaging in physical activity has been recommended as an important public health strategy to improve the health of children and adolescents. Nowadays, there is a decrease in physical activity and leisure time caused by computerization.^26^ ^,^ ^27^ In conjunction with the results demonstrated in this study, research has identified an association between the lowest levels of physical activity and internet addiction in adolescents.^28^ ^,^ ^29^ ^,^ ^30^ Also Babey et al.^28^ state that teenagers are likely to replace physical activity in their spare time with time on the computer.

The teenagers who go out to dance in nightclubs or shows showed greater dependence on the internet. This result corroborates the argument of Zhou et al.,^9^ who inferred that internet usage contributes to maintaining or increasing social commitments. Usually, teenagers attend shows and nightclubs as part of a social time. Thus, it is believed that they experience real time in connection with the virtual space of the internet, using it for their personal promotion in search of self-affirmation.

As an adjustment variable in the modeling, the weekly habit of reading a newspaper, magazine or book is an indicator of protection against internet dependence. In this sense, Sasmaz et al.^24^ affirmed that the construction of a healthy living environment through the control of computer use and internet access, and the promotion of book reading, favor the prevention and treatment of internet addiction in adolescents.

There are some limitations in this study that should be highlighted. First, the cross-sectional design used in the present study made it impossible to identify the causality of factors associated with internet dependence. Second, the data were obtained from a self-administered questionnaire by the adolescents, with no complementary information from parents or other informants.

In conclusion, the present study showed a prevalence of 10.6% for internet addiction in a sample of high school adolescents in Acre. Female adolescents were more susceptible to internet addiction when compared to male adolescents. Time spent on the computer and physical inactivity were major factors for internet dependence. From the leisure time activities, the habit of reading proved to be indicative of being protective for internet dependence. On the other hand, attending shows and nightclubs was positively associated with internet dependence.

In view of this evidence, for the prevention of internet addiction in schoolchildren, implementing strategies aimed at reducing internet time and computer use at school and in a social-family context are recommended. Concomitantly, encouraging the participation in physical activities and in reading newspapers, magazines and books is suggested.
